# 650 nm red-light therapy attenuates sepsis-induced acute lung injury via adiponectin-mediated immune–metabolic reprogramming

**DOI:** 10.3389/fimmu.2026.1710363

**Published:** 2026-01-22

**Authors:** Yiqiu Zhang, Wei Ni, Jianghan Li, Yubing Bai, Yier Bai, Zhixuan Jiang, Jiadie Wang, Yu He, Yafeng Li, Jing Yuan, Min Yao

**Affiliations:** 1 Department of Plastic and Reconstructive Surgery, Shanghai Ninth People’s Hospital, Shanghai Jiao Tong University School of Medicine, Shanghai, China; 2 Ministry of Education (MOE) Key Laboratory for Biomedical Photonics, Huazhong University of Science and Technology, Wuhan, China; 3 Department of Dermatology, Xinhua Hospital Affiliated to Shanghai Jiaotong University School of Medicine, Shanghai, China

**Keywords:** adiponectin, mitochondrial function, monocyte/macrophage, photobiomodulation, sepsis-induced ALI

## Abstract

**Background:**

Sepsis-induced acute lung injury (ALI) is driven by dysregulated innate immunity and mitochondrial dysfunction. Monocyte/macrophage trafficking and polarization critically shape disease trajectory, yet clinically tractable immunometabolic interventions are limited. We hypothesized that 650 nm red-light photobiomodulation (PBM) alleviates septic ALI by reprogramming myeloid responses and preserving mitochondrial function via adiponectin signaling.

**Methods:**

Septic ALI was induced by cecal ligation and puncture (CLP) in mice. Animals received 650 nm PBM (10 min, every 6 h, three times within 24 h). Survival, lung edema, histology, and serum cytokines were assessed. Lung chemokines/cytokines were profiled by 23-plex Luminex. Immune composition was analyzed by flow cytometry, and CCR2^+^/CX3CR1^+^ subsets were visualized in CcrRFP–Cx3cr1GFP mice using 3D cryo-fMOST. IHC quantified CX3CR1, CCR2, CD68, CD86, and CD206. Adiponectin was measured in serum/BALF and lung. Pathway relevance was tested by AdipoR1 siRNA. In LPS-stimulated RAW264.7 macrophages, PBM effects on cytokines, ATP, mitochondrial ROS (MitoSOX), membrane potential (JC-1), and MitoTracker fluorescence were evaluated, with/without AdipoR1 knockdown.

**Results:**

PBM prolonged survival, reduced lung edema, improved histopathology, and lowered systemic TNF-α, IL-6, IL-1β, and MCP-1. Luminex showed broad suppression of pro-inflammatory mediators (e.g., G-/GM-CSF, IL-1 family, IL-6, IL-12, IL-17A, TNF-α) and chemokines (CCL11, CXCL1, MCP-1/CCL2, CCL3/4/5), with increases in IL-4/IL-10/IL-13. Flow cytometry revealed decreased neutrophils, monocytes, and inflammatory macrophages, alongside restored eosinophils and resident macrophages. Cryo-fMOST and IHC demonstrated reduced CCR2^+^/CD86^+^ inflammatory cells and enrichment of CX3CR1^+^/CD206^+^ reparative cells. PBM elevated adiponectin in serum, BALF, and lung; AdipoR1 knockdown abrogated anti-inflammatory effects and myeloid rebalancing. *In vitro*, PBM dose-dependently suppressed LPS-induced TNF-α/IL-6 and IL-1β while increasing IL-10, restored ATP, reduced mitochondrial ROS, and improved membrane potential, that benefits lost with AdipoR1 silencing.

**Conclusions:**

Septic ALI modulated by 650 nm PBM was characterized by suppressing CCR2^+^ inflammatory recruitment, enriching CX3CR1^+^/M2-like macrophages, and preserving mitochondrial function through adiponectin–AdipoR1 signaling. These data position red-light PBM as a mechanistically grounded, non-invasive method for sepsis-associated lung injury.

## Introduction

Sepsis, defined as life-threatening organ dysfunction caused by a dysregulated host response to infection, remains a significant cause of morbidity and mortality worldwide. Among the various organs affected, the lungs are particularly susceptible, with sepsis-induced acute lung injury (ALI) and acute respiratory distress syndrome (ARDS) representing severe clinical manifestations. The pathophysiology of septic ALI is characterized by an uncontrolled inflammatory response in the pulmonary microenvironment, in which innate immune cells, especially monocytes and macrophages, play central roles (1, 2).

Circulating CCR2^+^ classical monocytes (Ly6C^hi^) are rapidly recruited to the lungs via chemokine signals such as MCP-1/CCL2 upon systemic infection. These cells differentiate into inflammatory macrophages (M1-like), which produce high levels of pro-inflammatory cytokines (e.g., TNF-α, IL-6, IL-1β), contributing to alveolar damage, pulmonary edema, and impaired gas exchange (3, 4). In contrast, CX3CR1^+^ non-classical monocytes (Ly6C^low^) and tissue-resident macrophages, which are generally anti-inflammatory and associated with tissue repair (M2-like), are depleted or functionally suppressed during sepsis. Therefore, the imbalance between inflammatory CCR2^+^ and reparative CX3CR1^+^ mononuclear phagocytes is considered a hallmark of immune dysregulation in septic ALI (5).

Among the key mediators linking light exposure to systemic effects, adiponectin, an adipokine secreted primarily by adipose tissue, has emerged as a crucial regulator of inflammation and metabolism. Adiponectin exerts anti-inflammatory effects by inhibiting NF-κB signaling, reducing cytokine production, and promoting M2 macrophage polarization (6, 7). It also maintains mitochondrial integrity, suppresses ROS generation, and enhances ATP synthesis in immune and non-immune cells through its receptors, AdipoR1 and AdipoR2 (8, 9). Although its protective role has been studied in metabolic and cardiovascular disorders, the relevance of adiponectin signaling in septic lung inflammation and its regulation by red-light remain largely unexplored. Adiponectin has recently emerged as an important immunometabolic regulator in sepsis. Recent studies highlighted its anti-inflammatory and mitochondrial-modulating properties, as well as its therapeutic potential in sepsis, while emphasizing that the upstream regulatory mechanisms and cell-type–specific actions of adiponectin remain incompletely understood (10). These knowledge gaps underscore the need to elucidate how adiponectin signaling is dynamically regulated during septic acute lung injury and which cellular compartments predominantly mediate its protective effects.

Photobiomodulation (PBM), using low-level red or near-infrared light, has been increasingly recognized as a non-invasive approach capable of modulating immune responses and inflammatory microenvironments. Recent studies have shown that PBM can attenuate inflammatory tissue damage by regulating immune cell activation and cytokine production in diverse inflammatory models (11, 12). Red-light, particularly at a wavelength of 650 nm, has been shown to reduce inflammation, improve mitochondrial function, and promote tissue regeneration in models of wound healing and neuroinflammation (13–15). However, whether PBM exerts protective effects in sepsis-induced acute lung injury through modulation of immune cell dynamics and adipokine signaling has not yet been elucidated.

This study aimed to investigate the therapeutic potential of 650 nm red-light in a murine model of septic ALI induced by cecal ligation and puncture (CLP). We assessed its effects on survival, lung pathology, cytokine profiles, and mononuclear phagocyte dynamics. Furthermore, we explored the hypothesis that red-light therapy enhances adiponectin expression, thereby exerting anti-inflammatory and mitochondrial protective effects associated with adiponectin–AdipoR1 signaling. Our findings provide new mechanistic insight into light-based immunomodulation and support the use of red-light as a non-invasive therapeutic modality for sepsis-associated lung injury.

## Materials and methods

### Mice treatment

Wild-type C57BL/6 mice were purchased from the Shanghai Laboratory Animal Center of Chinese Academy of Sciences. B6.129(Cg)-Cx3cr1^tm1Litt^Ccr2^tm2.1lfc^/JernJ mice (JAX#032127) were purchased from the Jackson Laboratory, which is also known as Ccr^RFP^Cx3cr1^GFP^ dual-reporter mice that express red fluorescent protein in Ccr2 positive cells and green fluorescent protein in Cx3cr1 positive cells. The Animal Ethics Committee of the Shanghai Ninth People’s Hospital approved the research study. These experimental mice (6–8 weeks old, male) were housed in a standard environment with specific pathogen-free facility at 22 (± 2) °C and 12h light/dark cycle.

Septic acute lung injury was performed by cecal ligation and puncture (CLP) as the previous protocol (16). Mice were anesthetized with Isoflurane inhalation. The lower left abdomen was shaved and disinfected with 75% ethanol. A laparotomy was made, followed by a 1-cm cut through the abdominal wall to expose the cecum. Approximately two-thirds of the cecum near the base was ligated with a 4–0 silk suture, and a perforation was made in the ligated segment using a sterile 21-gauge needle to allow slight extrusion of fecal material. Sham-operated controls underwent the same anesthetic and surgical procedures as the CLP group, including midline laparotomy and gentle exteriorization of the cecum, but without cecal ligation or puncture. The cecum was then returned to the abdominal cavity, and the incision was closed in the same manner as in CLP-operated mice. The weight of the wet/dry lung was measured. Lung injury scores were evaluated based on the extent of neutrophil infiltration, alveolar and interstitial edema, and pulmonary hemorrhage, with scores ranging from 1 to 4 (17). The assessment principle was based on the Modified Murine Sepsis Score (MSS), which includes seven components of appearance: level of consciousness, activity, response to stimulus, eyes, respiratory rate, and respiratory quality (18) (N = 5). Scores were measured by three independent investigators who were blinded to group allocation.

AdipoR1-targeting and negative control small interfering RNA (siRNA) lentiviruses (GenePharma, China) were administered intranasally (8 μg/g body weight, once daily for three consecutive days) prior to induction of the CLP model.

### Cell culture and transfection

RAW264.7 murine macrophages (Procell Life Science & Technology Co., Ltd, China) were cultured in DMEM (Gibco, USA) with 10% fetal bovine serum (Gibco, USA) and 1% penicillin-streptomycin (HyClone, USA) at 37°C with a humidified atmosphere of 5% CO_2_. RAW264.7 cells were treated with LPS (0.1 μg/ml) for 24 h to establish a septic model.

AdipoR1 and negative control small interfering RNAs (siRNA) (GenePharma, China) were transfected into RAW264.7 cells using Lipofectamine 3000 (Invitrogen, USA) according to the manufacturer’s instructions. The target sequences were 5’-GGCUAAAGGACAACGACUATT-3’ (*adipoR1*-siRNA) and 5’-UUCUCCGAACGUGUCACGUTT-3’ (siNC). Cells were harvested for experiments 24 h post-transfection.

### 650 nm laser irradiation

The mice were treated with a 650 nm continuous semiconductor InGaAlP laser (Shanghai Laser Technology Research Institute, China). According to our previous study, the irradiation parameters were set at 80 mW for 10 min, 3 *cm* of spot diameter and 4 J/cm^2^ of energy density (19). The laser was set 5 cm above the vertical of the mice to ensure that the thorax and abdomen were irradiated. The irradiance at the skin surface was calculated before each experiment using a power meter (Thorlabs, USA) to ensure dose accuracy and reproducibility. Illumination was started immediately after CLP surgery and irradiated 3 times every 6 hours.

RAW264.7 cells were irradiated with the same equipment (40 mW, 5 mW/cm^2^) for various time (0, 25 s, 125 s, 250 s, 375 s). The corresponding energy density was 0.125, 0.625, 1.25 and 1.875 J/cm^2^. Irradiation was performed three times at 6 h intervals after LPS stimulation. Cells were collected for analysis 24 h later.

### Histology and immunostaining

Mice lung tissues were collected 24 hours after CLP model with 4% paraformaldehyde, paraffin-embedded, and sectioned into 5-μm-thick section. Then these sections were stained with hematoxylin & eosin (HE).

For immunohistochemical staining, sections were deparaffinized with xylene and incubated in 3% hydrogen peroxide solution, thereby blocking endogenous peroxidase activity. Non-specific protein binding was blocked by incubation in 5% bovine serum. These sections were incubated overnight at 4°C with primary antibodies against CD68 (ab201340, Abcam), CD86 (ab23400, Abcam), CD206 (ab64693, Abcam), CX3CR1 (ab308614, Abcam), CCR2 (ab273061, Abcam), Adiponectin (ab22554, Abcam) and incubated with secondary antibodies for 2 h at room temperature. Then images were acquired using a light microscope (BX53 Olympus, Japan) (N = 5).

### Cytokine profiling

Mice blood samples and bronchoalveolar lavage fluid (BALF) were collected and centrifuged at 2500 rpm for 20 min at 4°C. Then supernatants were analyzed by ELISA kits for TNF-α, IL-6, IL-1β, MCP-1 (N = 6) and adiponectin (N = 5) (MTA00B, M6000B, MLB00C, MJE00B, MRP300, R&D Systems, USA) and measured using chromatometry (BioAssay Systems, USA).

Lung tissues were isolated from mice and analyzed via the Bio-Plex Pro Mouse Cytokine 23-plex (#M60009RDPD, Bio-Rad, USA) based on the Luminex 200 system (Luminex Corporation, USA) following the manufacturer’s protocol. Briefly, the samples were incubated in 96-well plates for 30 min, following the detection antibody for 30 min including antibodies of CCL11, G-CSF, GM-CSF, IFN-γ, IL-10, IL-12p40, IL-12p70, IL-13, IL-17A, IL-1α, IL-1β, IL-2, IL-3, IL-4, IL-5, IL-6, IL-9, CXCL1, MCP-1, CCL3, CCL4, CCL5, TNF-α (N = 4).

### Flow cytometry

The lung tissue isolated from mice was minced and digested using collagenase for 1 h (N = 5). Then, obtain single-cell suspensions via cell strainers. The isolated immune cells were collected using the following antibodies purchased from BioLegend lnc., USA: anti-CD45 (clone S18009F), anti-CD11b (clone M1/70), anti-LY6G (clone 1A8), anti-CD11c (clone N418), anti-LY6C (clone HK1.4), anti-SiglecF (clone S17007L), anti-F4/80 (clone BM8). Isotype-matched control antibodies were used to assess nonspecific binding and to guide gate placement. In addition, fluorescence minus one (FMO) controls were included for key multicolor panels to assist in accurate identification of positive populations, particularly for markers with continuous expression. Following staining, cells were washed and acquired on a BD LSRFortessa flow cytometer (BD Biosciences, USA). Compensation was performed using single-stained controls. Data were analyzed using FlowJo software (TreeStar, USA). Gating strategies were applied consistently across all experimental groups.

Intracellular reactive oxygen species (ROS) were utilized with the fluorescent probe 2’,7’-dichlorodihydrofluorescein diacetate (DCFH-DA) (EEA019, ThermoFischer, USA) that was taken up by cells and converted to dichlorofluorescein (DCFH) by intracellular esterase.

DCFH was oxidized into strong fluorescent DCF. The amount of ROS depended on the fluorescence of DCF (Ex/Em 502/525 nm). According to the manufacturer’s guideline, the cells were added with DCFH-DA working solution and incubated at 37°C for 30 min. Then, centrifuge and collect cells with serum-free medium 2 times. The fluorescence intensity was detected using flow cytometry analysis under the condition of FITC.

Mitochondrial Superoxide (Mitosox) production was measured by MitoSox Red Dye (M36008, ThermoFischer, USA). Cells were seeded in 6-well plates with/without LPS and 650 nm irradiation, and added with 5 μl dye reagent, followed by 30 min of incubation at 37°C. Then, RIPA buffer was used to wash each well. Collect cells and transfer them into cytometer tubes with complete medium. Cells will be analyzed by flow cytometry using the FITC channel.

### 3D cryo-fMOST imaging

Cryo-fluorescence micro-optical section tomography (cryo-fMOST) imaging was performed to qualitatively visualize the three-dimensional spatial distribution of CCR2^+^ and CX3CR1^+^ cells within the lung tissue (20). Briefly, the lungs of Ccr^RFP^Cx3cr1^GFP^ dual-reporter mice were perfused with PBS followed by 4% PFA (Sigma-Aldrich, USA). Mouse lung organs were excised and fixed for 24 h after 4% PFA at 4°C, then rinsed, dehydrated, and embedded in OCT (4583, Sakura Finetek USA Inc.). The cryo-fMOST system imaged the cryogenic whole lungs.

### RNA-sequencing and reverse transcription-quantitative PCR

The total RNA was extracted from lung tissues using Trizol reagent (Thermofisher, USA). NanoPhotometer spectrophotometer (IMPLEN, USA) measured the purity and quantity. High-quality mRNA was constructed into the library and reverse-transcribed into PCR-amplified cDNA. The average size of the cDNA library was 300 ± 50 bp, which was performed on an Illumina Novaseq platform. According to gene expression analysis, GO and Kyoto Encyclopedia of Genes and Genomes (KEGG) enrichment were carried out.

The RNA of tissue and cells was isolated using Trizol reagent (Thermofisher, USA) and reverse-transcribed using the PrimeScript RT Master Mix kit (Takara, Japan). PCR primers were synthesized by the Sangon Biotechnology Company (China), as shown in **Table 1**. The gene expression was quantified by using the SYBR Green kit (Qiagen, USA). The relative changes were calculated using a 2^−ΔΔCt^ method and normalized to Gapdh.

**Table 1:** Lists of primers sequences of RT-qPCR.

Gene	Forward (5′–3′)	Reverse (5′–3’)
*Tnf-α*	CCACCACGCTCTTCTGTCTA	CTCCTCCACTTGGTGGTTTG
*Il-6*	GGAGAGGAGACTTCACAGAGGAT	AGTGCATCATCGCTGTTCATAC
*Adipoq*	CCAATGTACCCATTCGCTTTAC	GAAGTAGTAGAGTCCCGGAATG
*Il-1β*	TTGACGGACCCCAAAAGATG	AGAAGGTGCTCATGTCCTCAT
*Il-10*	GCTCTTACTGACTGGCATGAG	CGCAGCTCTAGGAGCATGTG
*gapdh*	CTCATGACCACAGTCCATGC	TTCAGCTCTGGGATGACCTT

### ATP assay

ATP content was analyzed using the CellTiter-Glo Luminescent Cell Viability Assay (Promega, USA). RAW264.7 cells were seeded in a 96-well plate at 1×10^4^ cells/well. After 24 h of treatment with LPS or 650 nm irradiation, 100 μl CellTiter-Glo buffer was added to each well using the opaque plate. Then shake the plates for 2 min to mix thoroughly and incubate it for 10 min at RT. The luminescence was recorded by a multifunctional microplate reader Infinite 200 (Tecan, Austria).

### Mitochondrial membrane potential

The green-fluorescent JC-1 probe is available as a monomer at low concentrations or with low membrane potential. However, at higher concentrations or higher potentials, JC-1 forms red fluorescent “J aggregates” with a broad excitation spectrum and a maximum emission wavelength of about 590 nm. Therefore, the emission of this cyanine dye can be used as a sensitive measure of mitochondrial membrane potential. Briefly, 3×10^6^ cells/ml were stained with 2 mM JC-1 dye (T3168, Thermofisher, USA) for 30 min at 37°C. Then remove the dye and wash the wells twice. Fluorescence images were acquired at 535/590 nm and 475/530 nm by confocal microscopy (LSM710, Carl Zeiss, Germany). Mitochondrial membrane potential (Δψm) was quantified by calculating the ratio of red fluorescence (JC-1 aggregates, indicating high Δψm) to green fluorescence (JC-1 monomers, indicating low Δψm) based on mean fluorescence intensity (MFI). Confocal images were analyzed using ImageJ software. For each experimental condition, at least 5 randomly selected fields were analyzed per replicate.

Cellular mitochondrial damage can be studied using MitoTracker deep red (M22426, Thermofisher, USA). MitoTracker dyes are a class of cell-permeable fluorescent probes based on sulfhydryl-reactive chloromethyl moieties that are specific for mitochondrial staining. Treated cells were stained with MitoTracker dye for 30 min at RT. Images were captured using confocal microscopy (LSM710, Carl Zeiss, Germany). Mitochondrial activity was quantified by measuring the mean fluorescence intensity (MFI) per cell using ImageJ. The same imaging fields and cell numbers were analyzed as described above to ensure consistency across conditions.

### Statistics

All experiments were performed in triplicate, and data were analyzed using GraphPad Prism 8.0 software. Results are presented as mean ± standard deviation (SD). Prior to statistical testing, data distribution was assessed for normality using the Shapiro–Wilk test. For comparisons between two groups, an unpaired two-tailed Student’s t-test was applied. For comparisons among multiple groups, one-way analysis of variance (ANOVA) followed by appropriate *post hoc* tests was used.

For multiplex cytokine analyses, p values were adjusted for multiple comparisons using the Benjamini–Hochberg false discovery rate (FDR) method. An adjusted p value < 0.05 was considered statistically significant. For RNA sequencing, differentially expressed genes were identified based on an absolute log2 fold change ≥ 1 and an FDR-adjusted p value < 0.05 using the Benjamini–Hochberg method. Survival curves were generated using the Kaplan–Meier method and compared using the log-rank (Mantel–Cox) test. A P value < 0.05 was considered statistically significant.

## Results

### 650 nm red-light alleviates pulmonary damage and mortality in septic ALI

**Figure 1:**
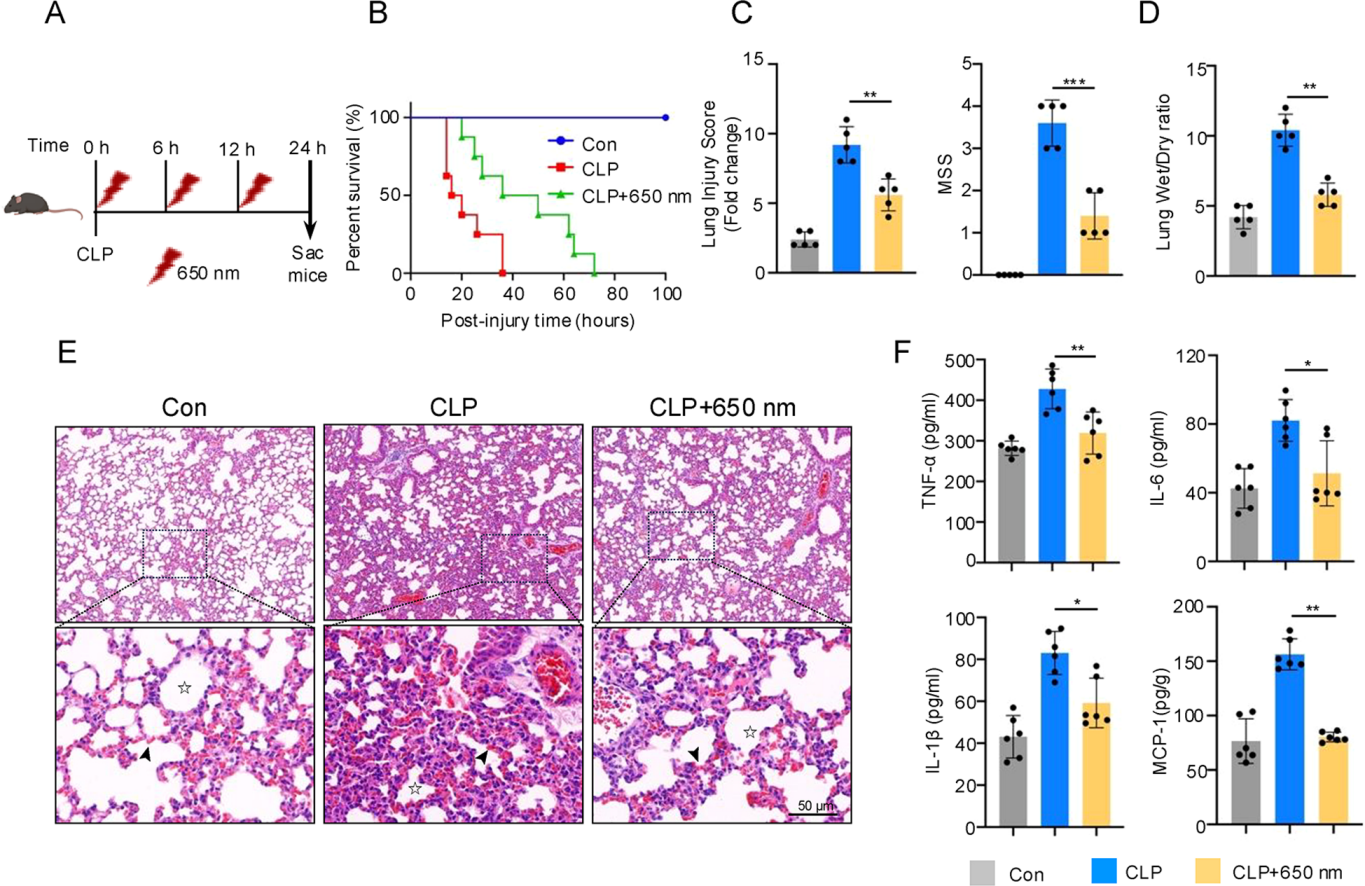
Protective effects of 650 nm photobiomodulation on septic ALI in mice. **(A)** Schematic illustration of the experimental timeline. **(B)** Kaplan-Meier survival curves of mice in the control, CLP, and CLP + 650 nm were analyzed using the Kaplan–Meier method with log-rank (Mantel–Cox) test. N = 8. **(C)** Lung injury scores and Murine Sepsis Score (MSS) based on histological and clinical examination. N = 5. **(D)** Lung wet/dry weight ratio. N = 5. **(E)** H&E staining of lung tissue sections (scale bar = 50 μm). Asterisks highlight alveolar structures; arrows indicate infiltrating red blood cells. **(F)** Levels of TNF-α, IL-6, IL-1β and MCP-1in the lung. N = 6. Data were analyzed by one-way ANOVA followed by Tukey’s *post hoc* test for multiple comparisons **(C, D, F)**. Data are presented as mean ± standard deviation. *p < 0.05 and **p < 0.01 versus the CLP group.

To evaluate the therapeutic effect of 650 nm red-light on septic acute lung injury (ALI), a cecal ligation and puncture (CLP) mouse model was established. Mice were treated with 650 nm red-light for 10 min with a 6 h interval and received three treatments within the first 24 h after CLP (**Figure 1A**). Initially, CLP mice exhibited classic signs of sepsis, including lethargy, reduced mobility, diminished responsiveness, excessive eye discharge, and rapid, labored breathing. Histological analysis demonstrated extensive inflammatory cell infiltration, destruction of alveolar architecture, and red blood cell congestion in alveolar capillaries in CLP mice. The red-light group substantially attenuated these pathological changes (**Figure 1E**). These clinical symptoms and histological examination were markedly improved in the red-light group (**Figure 1C**). Survival analysis revealed that CLP-induced mice began dying as early as 14 hours post-surgery, with all animals succumbing within 36 hours. In contrast, the earliest death in the red-light-treated group occurred at 20 hours, and survival was prolonged, with some mice living up to 72 hours (**Figure 1B**).

At 24 hours post-CLP, systemic and pulmonary conditions were assessed. Given that the lungs are among the most susceptible target organs in sepsis-induced systemic inflammatory response syndrome (SIRS), pulmonary edema was evaluated using the wet-to-dry (W/D) weight ratio. The W/D ratio significantly increased to 10.40 ± 1.14 in CLP mice but was reduced to 5.80 ± 0.84 following red-light treatment (**Figure 1D**). Furthermore, serum levels of pro-inflammatory cytokines TNF-α, IL-6, IL-1β, and MCP-1 were significantly decreased following irradiation (**Figure 1F**). These findings indicate that 650 nm red light effectively mitigates lung injury, suppresses systemic inflammation, and prolongs survival in a murine model of septic ALI.

### 650 nm red-light modulates cytokine profiles and immune cell composition in septic ALI

**Figure 2:**
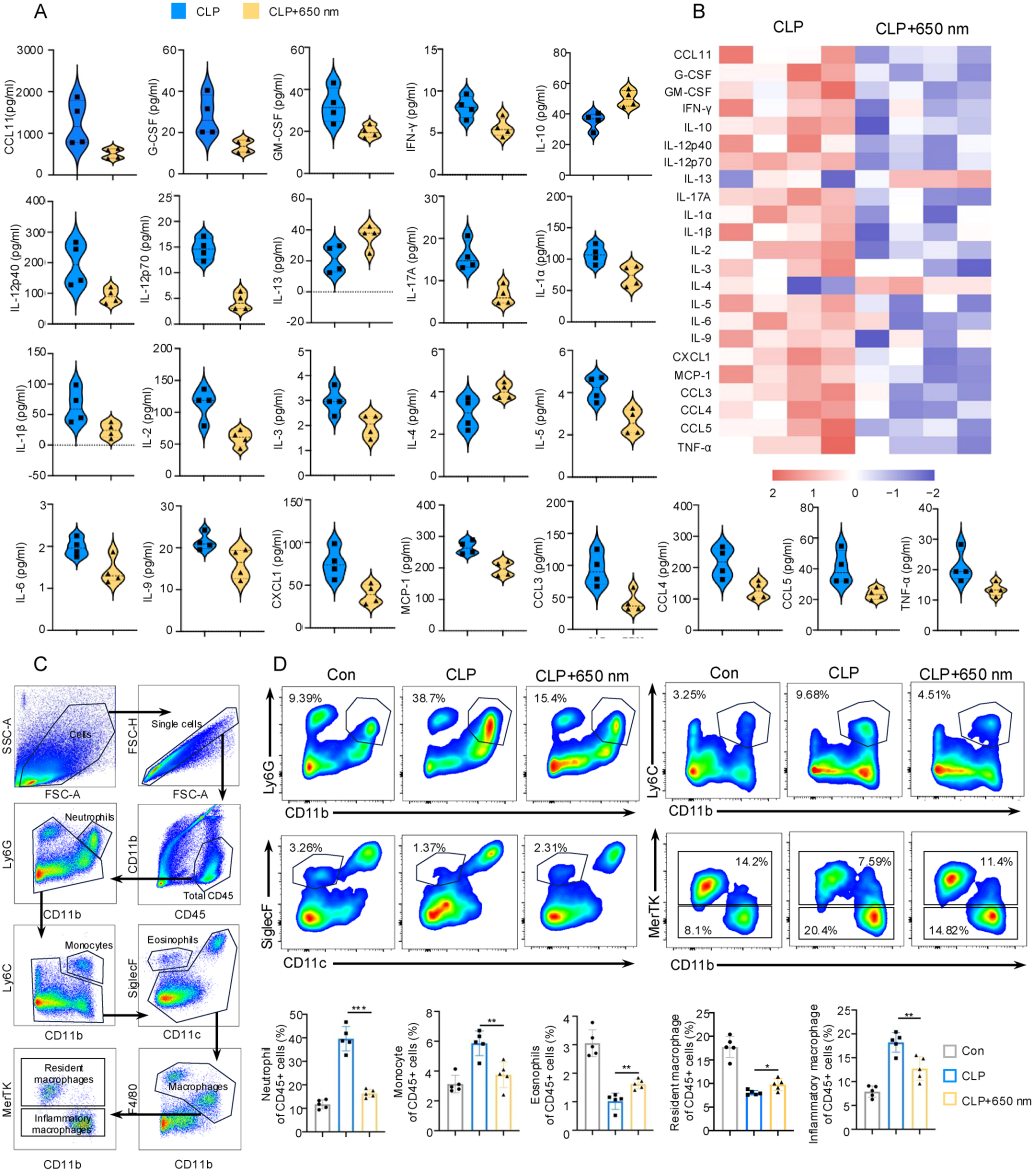
Modulation of pulmonary cytokine profile and immune cell infiltration by 650 nm photobiomodulation in septic ALI. **(A)** Violin plots showing the levels of various cytokines and chemokines in the lung from the CLP and CLP + 650 nm groups as determined by Luminex chip assay, N = 4. Data were performed using unpaired two-tailed Student’s t-tests with Benjamini–Hochberg false discovery rate (FDR) correction for multiple comparisons **(B)** Heatmap visualization transformed Luminex data, illustrating the relative expression levels of the measured cytokines. Each column represents an individual mouse. **(C)** Flow cytometry gating strategy for lung immune cell populations. **(D)** Flow cytometry analysis of immune cell populations in lung tissue. Representative contour plots and bar graphs showing the percentage of neutrophils (CD45^+^Ly6G^+^CD11b^+^), monocytes (CD45^+^Ly6C^+^CD11b^+^), eosinophils (CD45^+^SiglecF^+^CD11c^−^), resident macrophages (CD45^+^SiglecF^+^CD11c^+^MerTK^+^CD11b^−^), and inflammatory macrophages (CD45^+^CD11b^+^F4/80^+^MerTK^−^) within the CD45^+^ cell population, N = 5. Flow cytometry data were analyzed by unpaired Student’s t-test. Data are presented as mean ± standard deviation. *p < 0.05, **p < 0.01, and ***p < 0.001.

A 23-plex Luminex assay was performed on lung tissue samples to explore further the molecular mechanisms underlying red light’s anti-inflammatory effects. Following red-light treatment in CLP mice, 20 cytokines were downregulated while 3 were upregulated (**Figures 2A, B**). Notably, the levels of CCL11, CXCL1, MCP-1/CCL2, CCL3, CCL4, and CCL5 were significantly reduced, suggesting diminished recruitment of monocytes and other inflammatory leukocytes (T cells and NK cells) to the lung. Additionally, pro-inflammatory cytokines including G-CSF, GM-CSF, IL-12p40, IL-12p70, IL-17A, IL-1α, IL-1β, IL-2, IL-3, IL-5, IL-6, IL-9, and TNF-α were markedly suppressed, indicating reduced tissue infiltration and inhibition of monocyte and macrophages. In contrast, cytokines (IL-4, IL-10, and IL-13) associated with anti-inflammatory responses and tissue repair were upregulated following irradiation. While the downregulation of IFN-γ (a key driver of M1 polarization) was observed, its underlying mechanism remains unclear. These results suggest that 650 nm red-light may reduce immune cells especially monocyte infiltration by suppressing chemokine expression (e.g., MCP-1), thereby facilitating inflammation resolution and tissue regeneration.

Monocytes, originating from peripheral blood, migrate to the lung in response to inflammatory cues (e.g., IL-6, TNF-α), where they differentiate into inflammatory macrophages (M1) or dendritic cells, contributing to acute and chronic inflammation and tissue damage. In contrast, resident macrophages, derived from embryonic progenitors, play key roles in debris clearance, immune surveillance, and homeostasis maintenance. Eosinophils secrete anti-inflammatory factors such as IL-4, IL-10, and IL-13, which help inhibit excessive inflammation and promote tissue repair. Flow cytometric analysis of lung tissue was performed to characterize immune cell dynamics in response to red light (**Figures 2C, D**). Increased neutrophils, monocytes, and inflammatory macrophages were observed in the CLP group, alongside decreased eosinophils and resident macrophages. Strikingly, red light exposure reversed these alterations, significantly reducing neutrophils, monocytes, and inflammatory macrophages, while increasing eosinophils and resident macrophages. These findings demonstrated that sepsis could aggravate neutrophil and monocyte infiltration and inflammatory macrophage aggregation, inhibiting eosinophil and resident macrophages’ recruitment or survival. Red light may participate in immunomodulation and tissue repair by regulating the immune microenvironment (e.g., IL-6/IL-13).

### 650 nm red-light therapy reshapes pulmonary monocyte/macrophage subsets in septic ALI

**Figure 3:**
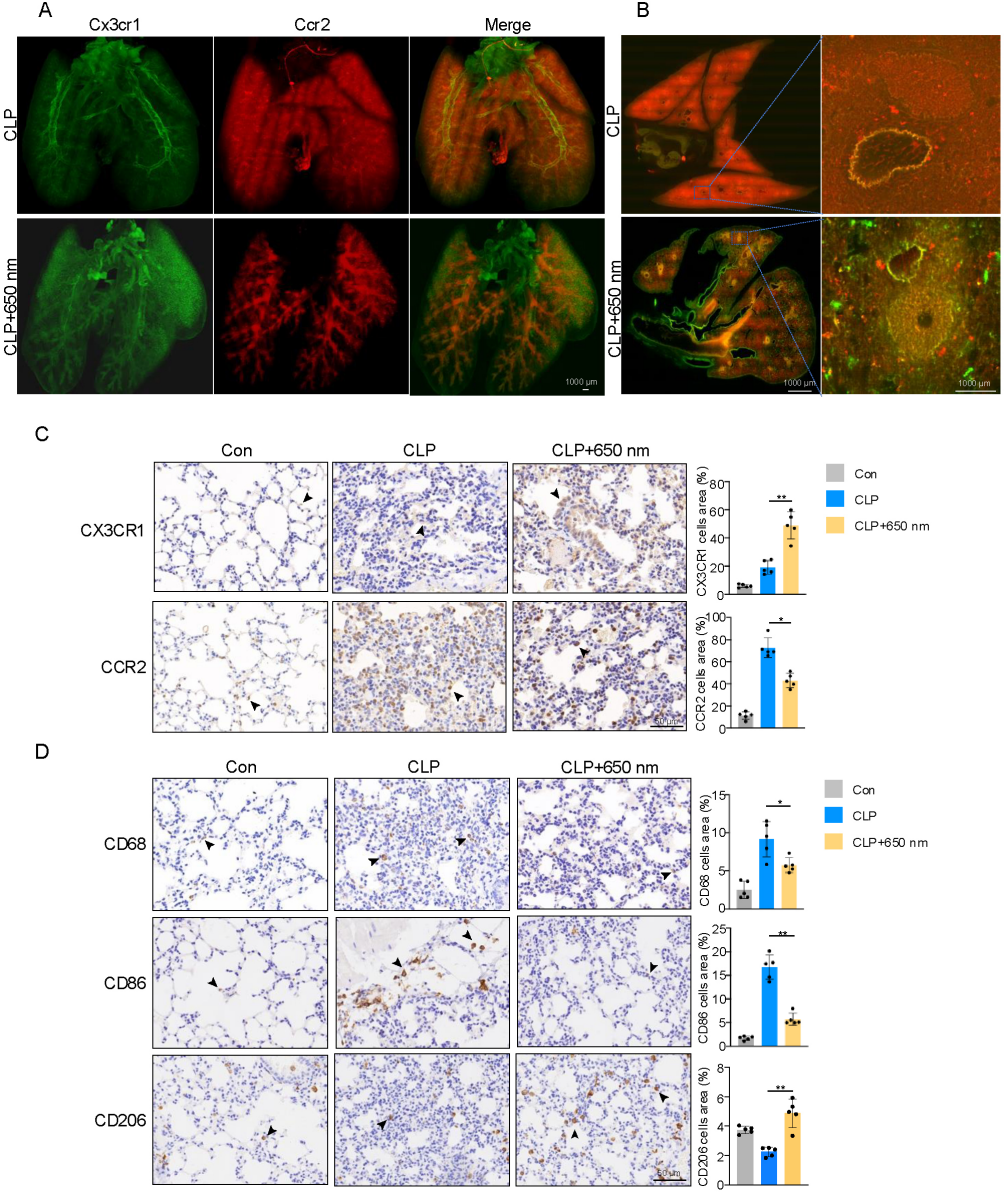
Red-light exposure modulates CX3CR1^+^/CCR2^+^ monocyte distribution and lung macrophage polarization during sepsis. **(A)** Representative whole-lung fluorescent images from Ccr^RFP^Cx3cr1^GFP^ dual-reporter mice at 24 h post-CLP with or without 650 nm red-light treatment. **(B)** Enlarged views of lung sections. Scale bars: 1000 μm. **(C, D)** Immunohistochemistry of lung tissues showing expression of CX3CR1, CCR2, CD68, CD86, and CD206 across control, CLP, and CLP + 650 nm groups. Scale bar = 50 μm. Arrows represent positive cells. N = 5. Quantitative immunohistochemistry data were analyzed using one-way ANOVA followed by Tukey’s *post hoc* test for multiple comparisons. *p < 0.05, **p < 0.01.

To further investigate the cellular origin of monocyte/macrophage alterations following red-light therapy, we employed Ccr^RFP^Cx3cr1^GFP^ dual-reporter mice to visualize the distribution of inflammatory and resident monocyte/macrophage subsets in lung tissue using 3D cryo-fMOST imaging. In the CLP group, fluorescence imaging revealed a predominance of CCR2^+^ (red) cells, indicative of robust infiltration by inflammatory monocytes, while CX3CR1^+^ (green) cells were relatively sparse. Upon treatment with 650 nm red-light, the abundance of CCR2+ cells markedly declined, particularly around vascular and pulmonary alveoli regions, whereas CX3CR1^+^ cell signal was noticeably increased (**Figure 3A**). Higher magnification views of lung lobes showed anatomical structures (**Figure 3B**). Large airways (bronchi) and pulmonary blood vessels can also be observed which often adjacent to bronchi and surrounded by dense infiltrates in the CLP group. Green signal was dramatically improved around blood vessels in light group. 3D imaging video in **Supplementary Videos 1** and **2**. CX3CR1^+^ and CCR2^+^ monocyte/macrophage subsets exhibit distinct phenotypic and functional characteristics that are critical in regulating inflammation and tissue homeostasis. CX3CR1 (C-X3-C chemokine receptor 1) is predominantly expressed on non-classical monocytes (Ly6C^low^) and tissue-resident macrophages, which are associated with anti-inflammatory and tissue-repair functions. These cells patrol the vasculature under steady-state conditions, maintain immune surveillance, and promote inflammation resolution. Elevated CX3CR1 expression is commonly linked to M2-like macrophages, suggesting a role in tissue remodeling and immune regulation.

In contrast, CCR2 (C-C chemokine receptor type 2) is highly expressed on classical monocytes (Ly6C^hi^), which are rapidly mobilized from the bone marrow in response to inflammatory stimuli such as CCL2 (MCP-1). CCR2^+^ monocytes infiltrate inflamed tissues and differentiate into inflammatory macrophages (often M1-like), contributing to the production of pro-inflammatory cytokines including TNF-α and IL-1β. These cells play a central role in amplifying the inflammatory response and pathogen clearance but may also exacerbate tissue injury when excessively activated. This phenotypic shift suggests that red-light significantly attenuates inflammatory monocyte recruitment while promoting the preservation or reconstitution of resident monocytes/macrophages. These findings provide *in vivo* evidence that red-light mitigates pulmonary inflammation by modulating monocyte/macrophage dynamics, suppressing M1-prone inflammatory infiltration, and potentially supporting M2-associated tissue repair.

To further characterize the phenotypic alterations of mononuclear phagocytes in the lung following red-light therapy, immunohistochemical staining was performed to evaluate the expression of CX3CR1, CCR2, CD68, CD86, and CD206 in lung tissues. As shown in **Figure 3C**, CX3CR1^+^ cells, which mark resident monocytes and patrolling macrophages, were markedly reduced in the CLP group compared to control mice, indicating depletion or dysfunction of the tissue-resident monocyte/macrophage population during sepsis. Red-light treatment significantly restored CX3CR1 expression levels, suggesting preserving or reconstituting the resident cell pool. In contrast, CCR2^+^ cell infiltration—a hallmark of inflammatory monocyte recruitment—was substantially increased in the CLP group but was notably attenuated following 650 nm light exposure. In parallel, we observed that CD68^+^ pan-macrophage populations were significantly elevated in CLP-induced septic lungs and remained elevated, though slightly reduced, upon red-light treatment. Notably, CD86 (an M1 marker) was strongly upregulated in the CLP group, reflecting a shift toward a pro-inflammatory macrophage phenotype. Red light treatment markedly suppressed this upregulation, indicating inhibition of M1 polarization. Conversely, CD206 (a canonical M2 marker) was significantly downregulated in the CLP group and partially restored after red-light exposure, suggesting a repolarization toward the M2 phenotype (**Figure 3D**).

These findings demonstrate that 650 nm red-light mitigates sepsis-induced pulmonary inflammation by rebalancing monocyte/macrophage subsets and reducing CCR2^+^ inflammatory infiltration while preserving or restoring CX3CR1^+^ resident cells, and by modulating macrophage polarization from an M1- to M2-like phenotype.

### 650 nm red-light suppresses pulmonary inflammation via modulating adiponectin expression

**Figure 4:**
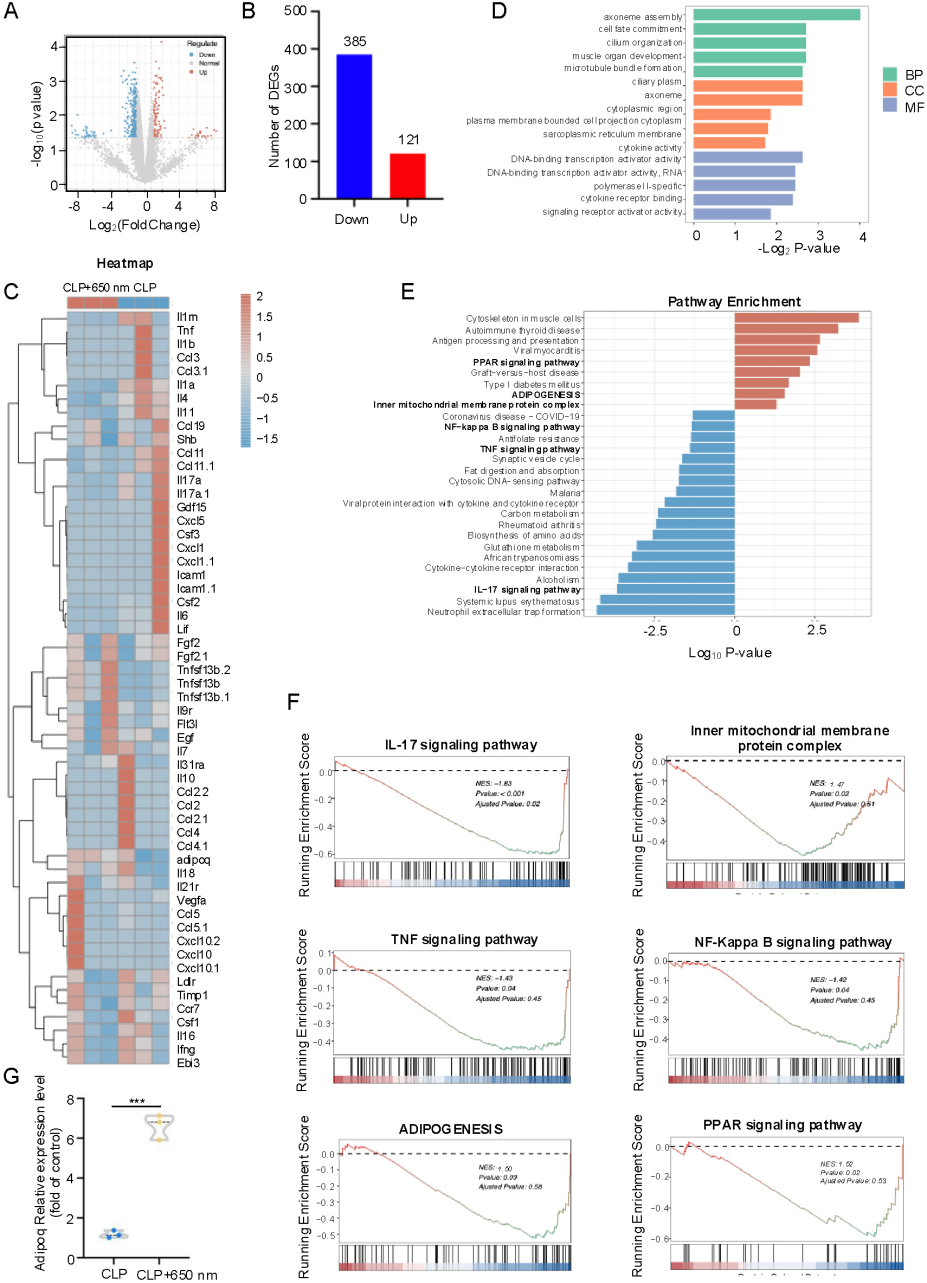
Transcriptomic alterations in lung tissue following 650 nm red-light treatment in septic mice. **(A)** Volcano plot of differentially expressed genes (DEGs). **(B)** Bar graph summarizing the number of significantly up-regulated and down-regulated genes (p < 0.05). **(C)** Heatmap showing representative inflammatory and immune-related gene changes. **(D)** Gene Ontology (GO) enrichment analysis of DEGs across biological processes, cellular components, and molecular functions. **(E)** Kyoto Encyclopedia of Genes and Genomes (KEGG) pathway analysis, with enriched pathways. **(F)** GSEA plots. Differentially expressed genes were identified using RNA-seq statistical models with Benjamini–Hochberg FDR correction. Genes with |log2 fold change| ≥ 1 and adjusted p < 0.05 were considered significant **(A–F)**. **(G)** RT-qPCR analysis of adiponectin mRNA expression in lung using unpaired Student’s t-test. ***p < 0.001.

To investigate the molecular effects of 650 nm red-light on lung tissue, we performed mRNA sequencing in CLP-induced septic mice. Volcano plot analysis revealed 385 downregulated and 121 upregulated genes following red-light treatment (**Figures 4A, B**). A heatmap highlighted broad suppression of inflammatory genes, including *Il6*, *Tnf*, *Il17a* and *Ccl2* family members, while *adipoq* was significantly upregulated (**Figure 4C**). GO analysis indicated enrichment of processes related to cytokine activity and cytokine receptor binding (**Figure 4D**), and KEGG/GSEA further identified IL-17, TNF, and NF-κB pathways as suppressed, with adipogenesis, PPAR signaling and Inner mitochondrial membrane protein complex enhanced (**Figures 4E, F**). Consistently, quantitative PCR analysis confirmed a robust increase of *adipoq* expression in red-light–treated lung (**Figure 4G**). These findings demonstrate that red-light profoundly reshapes lung transcriptomes, dampening pro-inflammatory signaling while promoting adiponectin expression and metabolic regulation.

**Figure 5:**
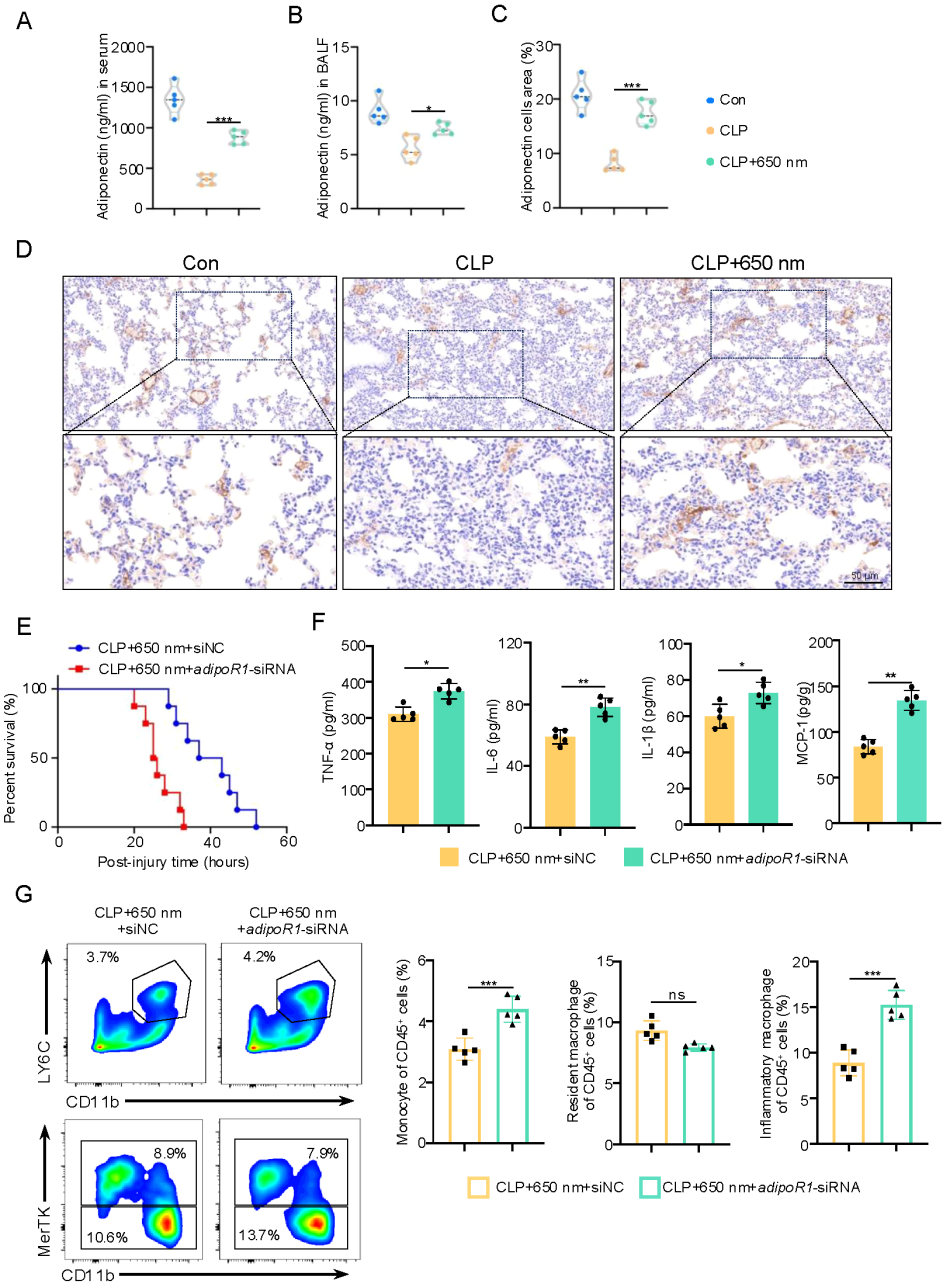
Red-Light (650 nm) elevates adiponectin and modulates pulmonary monocyte–macrophage dynamics. ELISA result showing adiponectin levels of serum **(A)** and BALF **(B)** (N = 3). **(C, D)** Immunohistochemical staining of adiponectin in lung sections and quantification of the adiponectin-positive cell area (N = 3). **(E)** Survival curves were analyzed using the Kaplan–Meier method with log-rank test (N = 8). **(F)** Serum TNF-α, IL-6, IL-1β, and MCP-1 levels in the CLP + 650 nm group with either siNC or adipoR1-siRNA (N = 5). **(G)** Flow cytometry analysis of lung tissue showing the percentage of monocytes (CD45^+^Ly6C^+^CD11b^+^), resident macrophages (CD45^+^SiglecF^+^CD11c^+^MerTK^+^CD64^+^CD11b^−^), and inflammatory macrophages (CD45^+^CD11b^+^F4/80^+^MerTK^−^CD64^+^) within the CD45^+^ cell population in the CLP + 650 nm group treated with siNC or adipoR1-siRNA (N = 5). These data were analyzed using unpaired Student’s t-test for two-group comparisons **(A–D, F, G)**. Data are presented as mean ± standard deviation. Scale bar = 50 μm. *p < 0.05, **p < 0.01, and ***p < 0.001.

Correspondingly, serum adiponectin levels were markedly elevated following red-light treatment (**Figure 5A**), suggesting a systemic upregulation of this protective factor. Consistently, BALF adiponectin concentrations were also significantly reduced in CLP mice but restored by red-light irradiation (**Figure 5B**). Immunohistochemical staining revealed the presence of adiponectin-positive cells in lung tissue sections. Quantitative analysis demonstrated a significantly increased percentage of adiponectin-positive cell area in the CLP + 650 nm group (17.47 ± 2.19%) compared to the CLP group (8.12 ± 1.54%) (**Figures 5C, D**). To determine the functional relevance of adiponectin signaling, we examined systemic inflammation after knockdown of adipoR1, the primary receptor mediating adiponectin’s effects. Importantly, intranasal delivery of siRNA markedly decreased survival in septic mice (**Figure 5E**), underscoring the protective role of adiponectin. The serum levels of TNF-α, IL-6, IL-1β, and MCP-1 were significantly increased compared to the control group (**Figure 5F**), indicating that disruption of adiponectin signaling exacerbates systemic inflammation. Furthermore, flow cytometric analysis of lung tissues (**Figure 5G**) showed that adipoR1 inhibition reversed the red-light–induced reductions in monocytes and inflammatory macrophages (M1), and decreased the proportion of resident macrophages. This shift in immune cell populations highlights the critical role of adiponectin signaling in regulating monocyte recruitment and macrophage polarization during sepsis. Taken together, these findings support the hypothesis that 650 nm red-light therapy exerts its anti-inflammatory effects, at least in part, through the upregulation of adiponectin. This enhancement of adiponectin appears to mitigate systemic and pulmonary inflammation by limiting the infiltration of pro-inflammatory immune cells such as monocytes and M1 macrophages, thereby promoting an anti-inflammatory and tissue-protective microenvironment.

### 650 nm red-light improves mitochondrial function and reduces inflammation in macrophages

**Figure 6:**
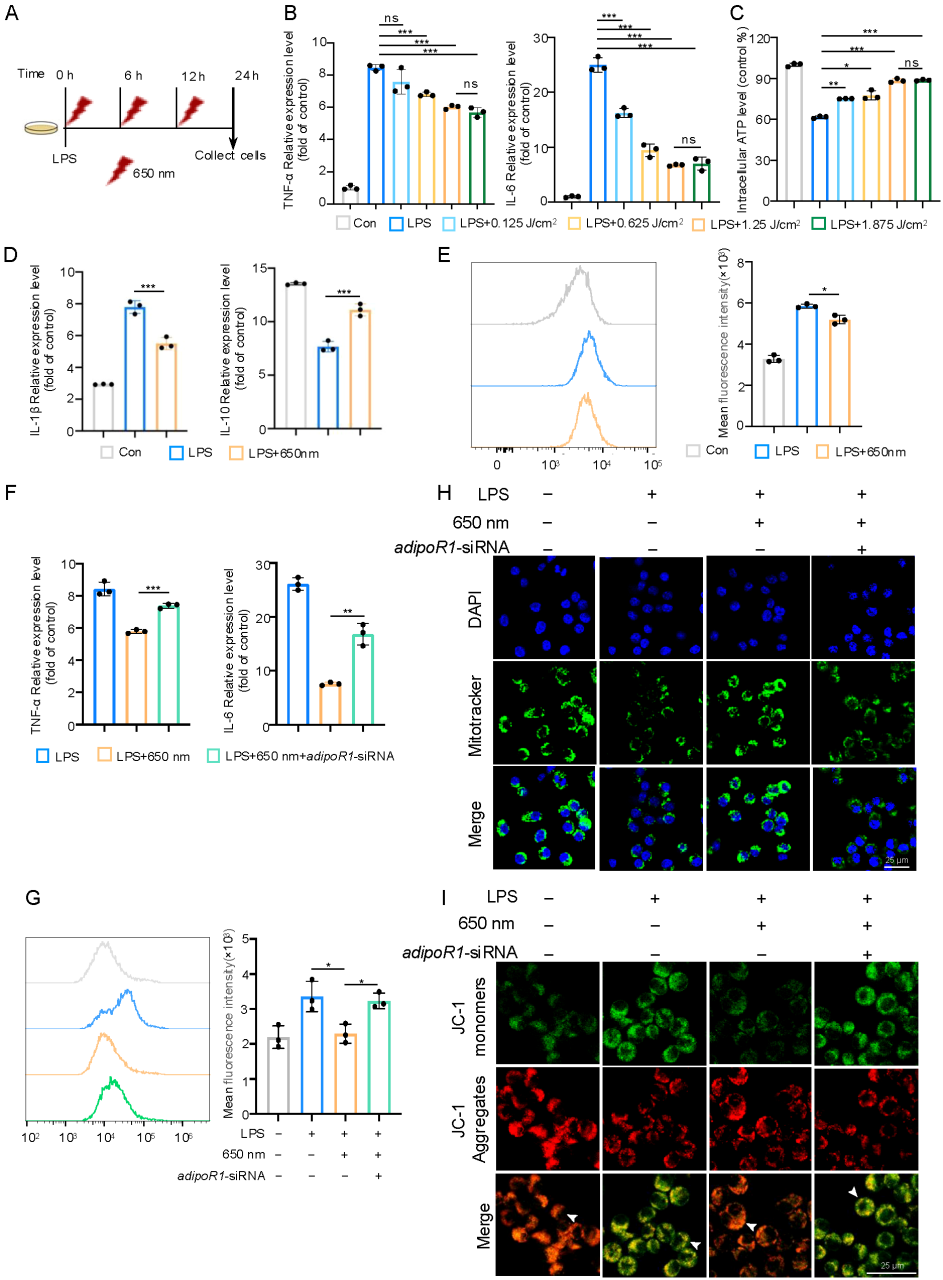
Photobiomodulation attenuates LPS-induced inflammation and oxidative stress in RAW264.7 macrophages via AdipoR1 signaling. **(A)** Schematic illustration of the *in vitro* experimental design. **(B)** Relative mRNA expression levels of TNF-α and IL-6 in LPS-stimulated macrophages treated with 650 nm light. **(C)** Intracellular ATP levels in LPS-stimulated macrophages treated with 650 nm light. **(D)** Relative mRNA expression levels of IL-1β and IL-10. **(E)** Flow cytometric analysis of mitochondrial superoxide production using MitoSOX and quantifying mean fluorescence intensity (MFI). **(F)** Relative mRNA expression levels of TNF-α and IL-6 in macrophages transfected with either siNC or adipoR1-siRNA. **(G)** Flow cytometric analysis showing intracellular ROS production. **(H, I)** Representative confocal microscopy images showing mitochondrial membrane potential (Δψm) using MitoTracker and JC-1 dye, and quantification of MFI. MitoTracker (green) and JC-1 aggregates (red) indicate high Δψm, while JC-1 monomers (green) indicate low Δψm. Nuclei are stained with DAPI (blue). Arrows represent merged cells. Data are presented as mean ± standard deviation. Data involving more than two groups were analyzed using one-way ANOVA followed by Tukey’s *post hoc* test **(B–E, H, I)**. Comparisons between two groups were performed using unpaired Student’s t-test **(F, G)**. Scale bar = 25 μm. *p < 0.05, **p < 0.01, and ***p < 0.001.

To explore the cellular mechanisms underlying the therapeutic effects of red-light, we established an *in vitro* sepsis model using macrophages and applied 650 nm red-light irradiation three times within 24 hours (**Figure 6A**). Macrophages were exposed to varying energy doses of red-light to evaluate changes in inflammatory cytokine expression and ATP production (**Figures 6B, C**). The results showed that LPS-induced expression of TNF-α and IL-6 progressively decreased with increasing light energy. However, no significant difference was observed between the 1.25 J/cm² and 1.875 J/cm² treatment groups. Similarly, while LPS suppressed ATP production in macrophages, red-light partially restored ATP levels, with no significant difference between the same two energy doses. We selected 1.25 J/cm² as the optimal irradiation parameter for subsequent experiments based on these observations. In addition, red-light treatment reduced LPS-induced IL-1β mRNA expression while increasing the anti-inflammatory cytokine IL-10 (**Figure 6D**), further supporting its role in promoting an anti-inflammatory macrophage phenotype. To further investigate mitochondrial function, MitoSOX staining revealed that LPS increased mitochondrial superoxide production, which was markedly reduced following red-light treatment (**Figure 6E**), suggesting that red-light alleviates mitochondrial oxidative stress.

We transfected macrophages with siRNA targeting adiponectin receptor 1 (adipoR1) to determine whether adiponectin contributes to these protective effects. Red-light significantly inhibited LPS-induced expression of TNF-α and IL-6, while silencing adipoR1 attenuated this inhibitory effect (**Figure 5F**), highlighting the involvement of adiponectin signaling in the anti-inflammatory actions of red-light. Regarding mitochondrial homeostasis, red-light effectively reduced mitochondrial ROS accumulation induced by LPS, an effect that was abolished by adipoR1 knockdown (**Figure 5G**). MitoTracker staining demonstrated increased mitochondrial membrane potential and activity following red-light treatment, as indicated by enhanced fluorescence intensity. This improvement was abrogated in cells transfected with adipoR1-siRNA, suggesting that red-light preserves mitochondrial integrity via adiponectin signaling (**Figure 5H**). Consistent findings were observed in JC-1 staining assays (**Figure 5I**). JC-1 accumulates in mitochondria as red-fluorescent aggregates under normal membrane potential, whereas it remains green-fluorescent monomers when the membrane potential is reduced. Red-light increased the red/green fluorescence ratio in LPS-treated macrophages, indicating restoration of mitochondrial membrane potential. However, adipoR1 knockdown reversed this effect, reducing red fluorescence and increasing green fluorescence, thus diminishing the mitochondrial protective effects of red-light.

These findings demonstrate that 650 nm red-light effectively suppresses inflammatory cytokine production and improves mitochondrial function in LPS-stimulated macrophages. The anti-inflammatory and mitochondrial protective effects are strongly associated with adiponectin–AdipoR1 signaling. Interruption of this pathway reverses the beneficial outcomes of red-light therapy, highlighting adiponectin as a critical mediator in modulating macrophage activation and maintaining mitochondrial homeostasis under septic conditions.

## Discussion

Sepsis-induced acute lung injury (ALI) remains one of the most devastating complications of systemic infection, characterized by excessive immune activation, cytokine storms, and profound mitochondrial dysfunction (21). Our study reveals that 650 nm red-light therapy (photobiomodulation, PBM) alleviates lung injury and systemic inflammation in septic mice by modulating monocyte/macrophage dynamics, restoring mitochondrial function, and enhancing adiponectin signaling. These findings deepen our understanding of immune-metabolic interactions in sepsis and introduce a non-invasive therapeutic strategy for modulating systemic immunity.

Monocytes and macrophages are central to the pathogenesis of ALI. Upon endotoxin stimulation, classical inflammatory monocytes (Ly6C^hi^, CCR2^+^) are rapidly mobilized from the bone marrow and infiltrate the lungs, where they differentiate into M1-like macrophages and perpetuate inflammatory tissue damage through secretion of TNF-α, IL-6 (22). In contrast, non-classical monocytes (Ly6C^low^, CX3CR1^+^) and M2-like macrophages are involved in immune resolution, tissue remodeling, and maintenance of lung homeostasis (23). The balance and plasticity between these subsets, CCR2^+^/CX3CR1^+^ and M1/M2, play a decisive role in determining whether the lung progresses toward resolution or sustained injury (24). Our study demonstrates that 650 nm red-light therapy significantly reshapes this cellular balance by reducing the infiltration of CCR2^+^ pro-inflammatory monocytes and promoting the enrichment of CX3CR1^+^ monocyte subsets and M2-like macrophages in the lung under 3D cryo-fMOST imaging. This phenotypic reprogramming coincides with reduced cytokine levels and histological improvement in alveolar architecture. These findings align with emerging concepts that monocyte/macrophage transitions are not merely passive consequences but active drivers of sepsis progression and resolution.

Notably, while previous studies have described the role of PBM in modulating macrophage phenotype, most have focused on *in vitro* settings or localized tissue contexts. For example, PBM has been shown to drive macrophages toward an anti-inflammatory (M2) phenotype by modulating mitochondrial ROS, calcium flux, and downstream NF-κB or STAT6 pathways (25–27). In contrast, our study employs transcutaneous PBM induces profound immunomodulation in the lungs of septic mice. This provides compelling *in vivo* evidence that PBM may exert systemic effects through circulating mediators or neuroimmune pathways.

Adiponectin is classically a systemic adipokine predominantly secreted by white adipose tissue, although local production by non-adipocyte cell types has been reported under inflammatory conditions. In this study, adiponectin levels were increased in serum, bronchoalveolar lavage fluid, and local organ following 650 nm photobiomodulation, suggesting contributions from both systemic and local pools (28). Increasing evidence shows its pivotal immunoregulatory functions. It inhibits macrophage activation, suppresses NF-κB, and promotes M2 polarization via AMPK, PPARγ, and STAT3 signaling axes (29, 30). Moreover, adiponectin enhances mitochondrial biogenesis and respiration by activating AMPK-PGC1α pathways, protecting against oxidative injury and energy failure (31). Here, we show that 650 nm red-light stimulates adiponectin expression in circulation and lung tissue, thereby contributing to the modulation of macrophage phenotype and systemic immune balance. Although adiponectin signals through both AdipoR1 and AdipoR2, the present study focused on AdipoR1, which is the predominant receptor implicated in macrophage-mediated inflammatory and metabolic regulation. AdipoR2, which has been more closely linked to lipid metabolism and PPAR-related signaling, was not directly evaluated in this study. Therefore, our mechanistic conclusions are restricted to AdipoR1-dependent pathways. The potential contribution of AdipoR2 to photobiomodulation-mediated effects during sepsis remains to be explored in future studies.

In the broader context of PBM research, increasing attention has been directed toward its systemic regulatory effects beyond local anti-inflammatory action. Studies in sepsis have demonstrated that PBM can reduce tissue oxidative stress, inhibit pro-inflammatory cytokine cascades, and preserve mitochondrial structure (32). Our results agree with these findings but extend them by identifying a specific immunometabolic pathway of adiponectin-mediated monocyte/macrophage reprogramming, as a novel mechanism of PBM action. This mechanistic insight not only deepens our understanding of PBM but also provides a concrete molecular target that could be further explored for biomarker development or combinatorial therapy. Furthermore, PBM significantly preserved mitochondrial integrity in macrophages. Mitochondrial homeostasis influences macrophage polarization: fragmentation promotes pro-inflammatory M1 phenotypes, while elongation and efficient oxidative phosphorylation support anti-inflammatory M2 phenotypes. Thus, PBM may confer dual benefits in septic ALI by suppressing inflammation and restoring cellular energy balance.

Despite the promising findings presented in this study, several limitations should be acknowledged. Although we demonstrate systemic immunomodulatory effects of 650 nm PBM, the precise mechanisms by which red light induces adiponectin expression in circulation and lung tissue remain incompletely defined, and direct causal links between light exposure, adiponectin signaling, and immune metabolism will require further validation using conditional or cell-specific genetic models. In addition, the use of young male mice, a single sepsis model, and a single PBM wavelength and parameter set may limit generalizability. *In vitro* experiments relied on the RAW264.7 macrophage cell line rather than primary alveolar macrophages. Finally, PBM was administered during the early phase of sepsis, with analyses focused on acute responses within 24 h. Longer-term outcomes and extra-pulmonary organ effects were not assessed.

**Figure 7:**
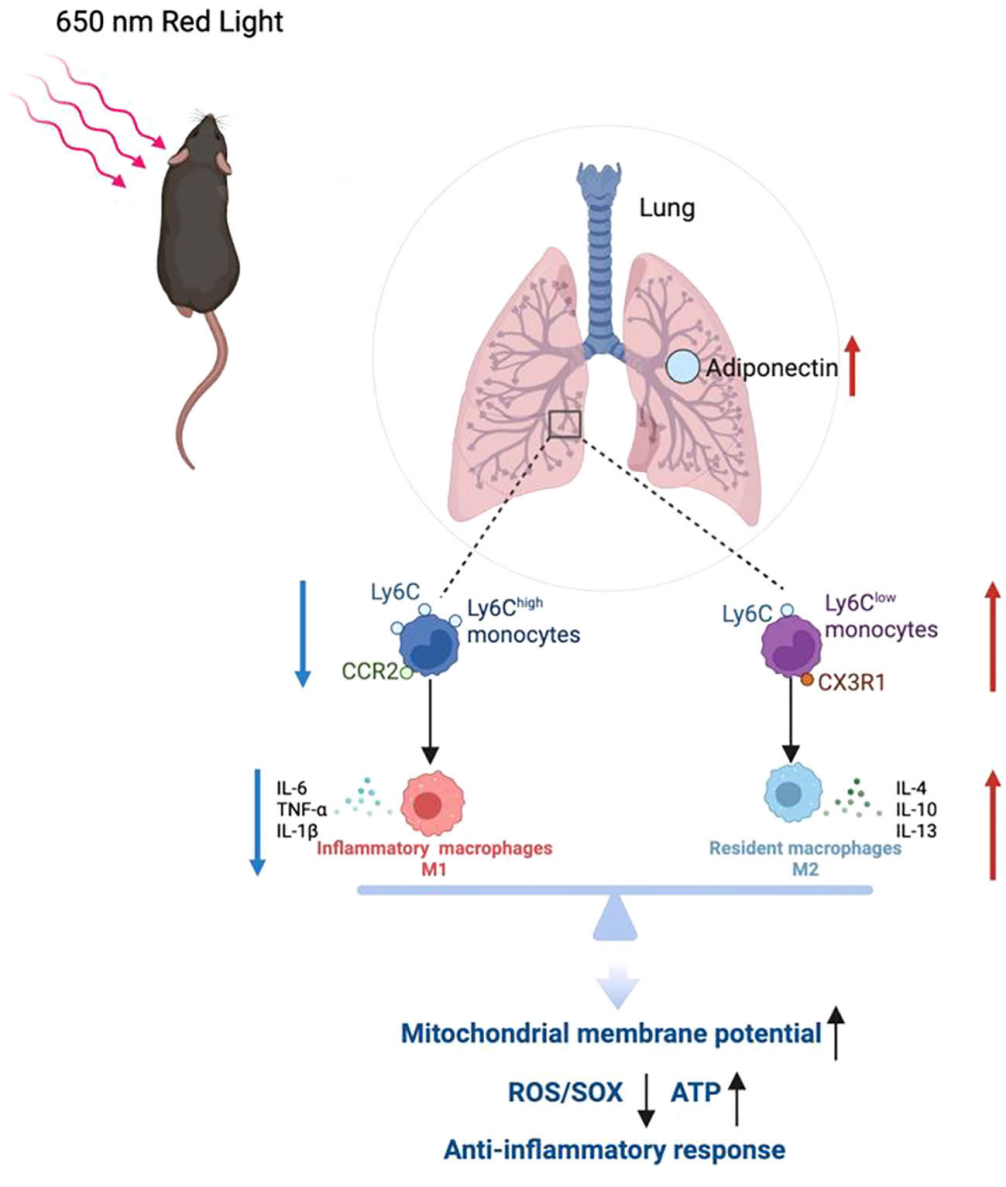
Mechanism of 650 nm red-light attenuation of sepsis-induced acute lung injury. 650 nm red-light irradiation stimulates adiponectin production, which, via the AdipoR1 signaling pathway in the lungs, modulates monocyte/macrophage recruitment and polarization (decreases CCR2^+^, increases CX3CR1^+^), exerts immunomodulatory effects (reduces inflammatory cytokines TNF-α, IL-6, IL-1β), and provides mitochondrial protection by improving mitochondrial function (increases ATP, reduces ROS/MitoSOX, maintains Δψm), ultimately attenuating sepsis-induced ALI.

In conclusion, our study reveals a multi-layered mechanism by which 650 nm red-light therapy alleviates sepsis-induced lung injury: (1) by reducing recruitment of CCR2^+^ pro-inflammatory monocytes and promoting CX3CR1^+^ and M2-like reparative cells; (2) by restoring mitochondrial integrity and reducing oxidative damage; and (3) by enhancing systemic adiponectin levels, which act as upstream regulators of immune and metabolic balance (**Figure 7**). The therapy of red-light is both non-invasive and easily scalable, providing mechanistic and proof-of-concept evidence supporting photobiomodulation as a promising experimental immunomodulatory strategy for sepsis-associated lung injury. This work identifies an adiponectin-dependent immunometabolic axis linking red-light exposure to systemic and pulmonary protection in sepsis, offering a novel strategy with potential translational relevance, warranting further validation in additional preclinical and clinical studies.

## Data Availability

The datasets presented in this study can be found in online repositories. The names of the repository/repositories and accession number(s) can be found below: PRJNA1333651 (SRA).

## Supplementary material

Supplementary Video 1 3D visualization of cleared lung tissue showing Cx3cr1^+^ (green) and Ccr2^+^ (red) cells in CLP mice.

Supplementary Video 2 3D visualization of cleared lung tissue showing Cx3cr1^+^ (green) and Ccr2^+^ (red) cells in CLP + 650 nm red-light mice.
